# PCR melting profile (PCR MP) - a new tool for differentiation of *Candida albicans *strains

**DOI:** 10.1186/1471-2334-9-177

**Published:** 2009-11-11

**Authors:** Beata Krawczyk, Justyna Leibner-Ciszak, Anna Mielech, Magdalena Nowak, Józef Kur

**Affiliations:** 1 Gdańsk University of Technology, Chemical Faculty, Department of Microbiology, ul. Narutowicza 11/12, 80-952 Gdańsk, Poland

## Abstract

**Background:**

We have previously reported the use of PCR Melting Profile (PCR MP) technique based on using low denaturation temperatures during ligation mediated PCR (LM PCR) for bacterial strain differentiation. The aim of the current study was to evaluate this method for intra-species differentiation of *Candida albicans *strains.

**Methods:**

In total 123 *Candida albicans *strains (including 7 reference, 11 clinical unrelated, and 105 isolates from patients of two hospitals in Poland) were examined using three genotyping methods: PCR MP, macrorestriction analysis of the chromosomal DNA by pulsed-field gel electrophoresis (REA-PFGE) and RAPD techniques.

**Results:**

The genotyping results of the PCR MP were compared with results from REA-PFGE and RAPD techniques giving 27, 26 and 25 unique types, respectively. The results showed that the PCR MP technique has at least the same discriminatory power as REA-PFGE and RAPD.

**Conclusion:**

Data presented here show for the first time the evaluation of PCR MP technique for candidial strains differentiation and we propose that this can be used as a relatively simple and cheap technique for epidemiological studies in short period of time in hospital.

## Background

It is still generally accepted that *Candida albicans *is the major etiologic pathogen of candidiasis (candidemia, candidosis). Candidemia accounts for 8 to 15% of nosocomial bloodstream infections and *C. albicans *is the causative agent in 50 to 70% of the disseminated *Candida *infections [1,2]. Therefore, it is important to control *C. albicans *infections through early diagnosis and prevention of candidiasis, especially for hospitalized patients. Over the last several years, molecular methods have proven quite useful to study strain relatedness, allowing retrospective examinations of putative candidosis outbreaks and assessments of the epidemiological aspects of such outbreaks. Electrophoretic Karyotyping connected with Pulsed Field Gel Electrophoresis (EK-PFGE), Southern blot hybridization with the midrepeat sequence Ca3, PCR-Restriction Fragment Length Polymorphism (RFLP), macrorestriction analysis of genomic DNA followed by pulsed field gel electrophoresis (REA-PFGE), Amplified Fragment Length Polymorphism (AFLP), Polymorphic Microsatellite Locus Analysis (PMLA), analysis of the repetive sequences (RPSs) and RAPD method have all been used for strain typing of *C. albicans *[3-13]. A very popular for fungal typing is multilocus sequence typing (MLST). The method is highly reproducible, and data can be archived in Web-based databases accessible to all users. For *C. albicans*, an MLST system based on seven DNA fragments was developed as an optimal consensus for typing strains within the species [14].

In general, there is a lack of consensus on which method (-s) to choose, as well as on how to interpret the results. The use of a single method may not be optimal, and a combination of typing techniques is often required to provide a comprehensive assessment of the epidemiology of candidiasis [15,16]. With this in mind, many laboratories are searching for a method that can provide the appropriate level of discriminatory power and is relatively rapid and cheap, especially for a large number of isolates.

**Figure 1:**
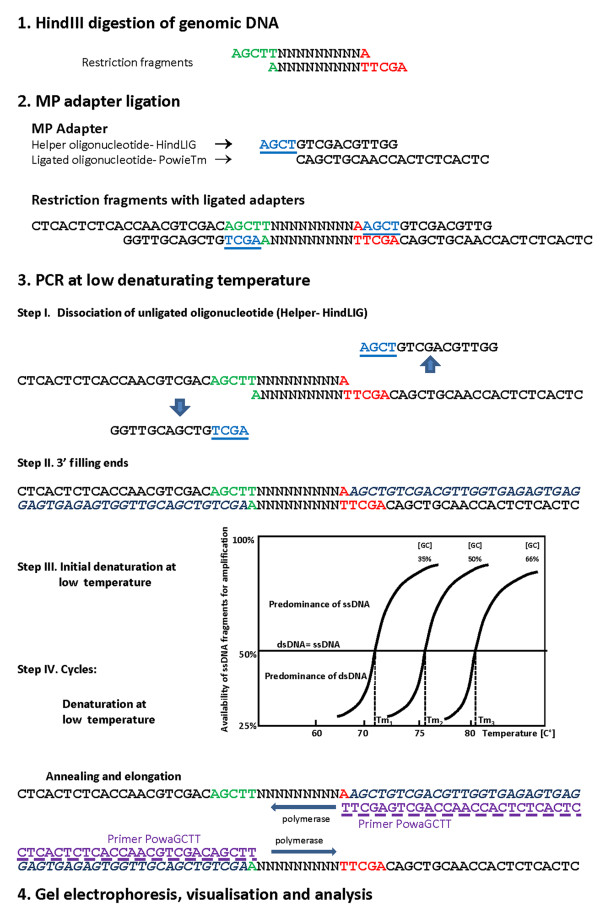
**Diagram illustrating the PCR MP technique**.

In this study, we demonstrate for the first time the use of a modified and optimized PCR Melting Profile (PCR MP) technique based on using low denaturation temperatures during ligation mediated PCR (LM PCR) for differentiation between *Candida albicans *isolates. This technique was developed by Masny and Plucienniczak [17] and Krawczyk *et al*. [18] for bacterial strain differentiation. Generally, in PCR MP a genomic DNA is completely digested with restriction enzyme and the restriction fragments are ligated with a synthetic adapter. During PCR, all DNA fragments in the sample should be amplified, however, lowering denaturation temperature (*T*_d_) in PCR decrease the number of amplified fragments due to that only single-stranded DNA may serve as a template for DNA synthesis. This allows gradual amplification of the DNA fragments differing in the thermal stability starting from the less stable DNA fragments amplified at lower denaturation temperature (*T*_d_) values to more stable ones amplified at higher *T*_d _values (Fig. 1).

We evaluated the typeability, reproducibility and discriminatory power of this technique in comparison with RAPD and REA-PFGE when used to distinguish between 123 strains of *C. albicans *from different geographic origins and patients.

## Methods

### Clinical specimens and *Candida strains*

In the current study the strain collection encompassed 123 strains of *C. albicans *(Table 1). These strains were divided into three groups. The first group consisted of 7 reference *C. albicans *strains and 11 strains from a collection kept at the Gdańsk University of Technology (unrelated strains, DM collection, Table 1). It was assumed that these strains were epidemiologically unrelated, based on detailed biochemical, clinical and epidemiological data. They were isolated over a significant period of time and were from different geographic origins (from patients in different Polish towns).

**Table 1:** Typing results of *C. albicans *strains using RAPD, REA-PFGE and PCR MP methods.

Name of strain	REA-PFGE Genotype	RAPD Genotype	PCR MP Genotype
***C. albicans *reference strains and clinical unrelated strains from Department of Microbiology collection (DM)**			
ATCC14053	1	1	1
ATCC10231	2	2	2
ATCC90029	3	3	3
ATCC64544	4	4	4
ATCC64545	3a	3a	3a
ATCC64550	5	5	5
ATCC64547	6	6	6
DM1	7	7	7
DM2	8	8	8
DM3	7	7	7a
DM4	nt	7	7b
DM5	9	9	9
DM6	10	10	10
DM7	11	11	11
DM8	11a	11	11a
DM9	12	12	12
DM10	13	13	13
DM11	14	14	14

***Candida albicans *GDAŃSK**			
G1/1	9	9	9
G1/2	9	9	9a
G2	9	9	9b
G3	9	9	9a
G4	9a	9	9a
G5/1	nt	10	10
G5/2	10	10	10
G6	11	10a	10a
G7/1	11	9a	11
G7/2	11	9a	11
G8/1	11	9a	11a
G8/2	11	9a	11
G9	11	9a	11a
G10	11	9a	11a
G11	11	9a	11a
G12	11a	9a	11b
G13	9b	9b	12
G14	9a	9b	12
G15	nt	9	12a
G16	12	11	13
G17	13	12	14
G18	nt	12	14a
G19	14	13	15
G20	14	13	15a
G21/1	14	13	15a
G21/2	14	13	15a
G22	14a	14	15a
G23/1	15	15	16
G23/2	15	15	16
G24/1	15	15a	16a
G24/2	15	15a	16a
G25	14	13	15a
G26	14	13	15a
G27	14	13	15a
G28	14	13	15a
G29	15	15a	16
G30	15	15a	16
G31/1	15	15a	16
G31/2	15	12a	16
G32	16	16	17
G33	15	15a	16
G34	15	15a	16
G35	15	15a	16
G36/1	15	15a	16a
G36/2	15	15a	16a
G37	15	15a	16a
G38	17	17	18
G39	15	15a	16a
G40/1	17	17	18
G40/2	17	17	18
G41	17	17	18a
G42	16	16	17
G43	16	16	17
G44	16	16	17
G45	17	17	18a
G46	17	17	18a
G47	18	18	19
G48/1	18	18	19
G48/2	18	18	19
G49	nt	18	19a
G50	17	17	18a
G51	17	17	18a
G52	19	19	20
G53/1	19	19	20
G53/2	19	19	20
G54/1	19	19	20
G54/2	19	19	20
G55/1	17	17	18b
G55/2	17	17	18b
G56	17	17	18a
G57	17	17	18a
G58	17	17	18a
G59	17	17	18a
G60	20	19	21
G61	20a	19	21a
G62	20	19	21
G63	20	19	21b
G64	19	19	20
G65	19	19	20
G66	19	19	20a
G67	21	20	22
G68/1	17	17	18a
G68/2	17	17	18a
G69	20a	19	21a
G70/1	nt	20	22a
G70/2	21	20	22
G71/1	17	17	18a
G71/2	17	17	18a
G72/1	21	20	22a
G72/2	21	20	22a
G73/1	19	19	20b
G73/2	19	19	20b
G74	nt	21	23
G75	22	21	23
G76	22	21	23

***Candida albicans *SZCZECIN**			
S1/1	23	22	24
S1/2	24	23	25
S2	24	23	25
S3/1	24a	23a	25a
S3/2	24a	23a	25a
S4/1 (mother)	25	24	26
S6/1 (child)	25	24	26
S4/2 (mother)	25	24	26
S5 (father)	26	25	27
S6/2 (child)	25	24	26

Genomic DNA for 7 reference strains and 116 clinical isolates of *C. albicans *was analyzed. Strains with identical sizes and numbers of bands were considered genetically indistinguishable and assigned to the same type (marked by number); strains with banding patterns that differed by up to three bands were considered closely related and described as subtypes (marked by letters). nt-not typable.

The second group consisted of strains that were collected from February 2006 to September 2007 from patients at the Hematology Clinic of the Public Hospital No. 1 in Gdańsk. We included 95 isolates of *C. albicans *from 76 patients (Table 1). The strains were isolated from stool (37), sputum (30), throat (17), blood (7) and oral cavity (4). Thirty eight of those *C. albicans *strains, isolated from different clinical specimens (sputum, stool, oral cavity or blood) from nineteen patients (two strains each), were used to study genotype variations in a patient.

The third group of *C. albicans *strains was collected from individual patients at the Public Hospital No 1 in Szczecin, and it consisted of 10 isolates of *C. albicans *from six patients (Table 1). The strains were isolated from the oral cavity (8), urine (1), and a wound (1). Five of those *C. albicans *strains were isolated from members of one single family.

The isolates used in this investigation were collected for routine diagnostic procedures by microbiological laboratories at the public hospital in Gdańsk and Szczecin and then were provided to our laboratory. Since this was a retrospective and methodological study on the isolates already available with us, it was not put up for any clearance to an ethical committee. All the patient files are confidential and none could be identified.

### DNA extraction

The yeasts were cultured in YPD (1% yeast extract, 2% peptone K, and 2% dextrose) at 30°C for 24 h before the DNA extraction. DNA from each strain was isolated from 3 ml of 24 h culture with the Genomic Mini AX Yeast kit (A&A Biotechnology, Poland) or GeneMATRIX Yeast DNA Purification kit (Eurx, Poland) according to the manufacturers procedure. The DNA concentrations were measured using NanoDrop ND-1000 (Thermo) and were in range from 40 to 400 ng per microliter.

### REA-PFGE

PFGE was performed with the Bio-Rad's Instruction Manual and Application Guide. A single colony from a sub-culture from the primary plates was grown overnight at 30°C in YPD under aerobic conditions. DNA was digested overnight at 25°C with 20 U of *Sma*I (BioLabs New England) for 1/3 plug, and separated on 1% agarose gel (BioRad) using the CHEF Dr II System as described by Deplano et. al. [19]. Electrophoresis was carried out at 200 V with alternating pulses from 2 to 8 s for 12 h and then from 10 to 15 s pulse time gradient for 12 h. Electrophoresis was performed in a cold condition at 10°C. A lambda DNA ladder (Bio-Rad) and Pulse Marker (Sigma) was used as a molecular size marker.

### RAPD

Isolates were typed by the random amplified polymorphic DNA technique (RAPD) using two primers: 1247 and 1290 [20] (Table 2). The amplifications were performed with approximately 30-40 ng of *Candida *DNA in 25 μl of a reaction mixture containing 2.5 μl of reaction buffer (500 mM KCl and 100 mM Tris-HCl, pH 8.3; 2.5 μl 25 mM MgCl_2_), 2.5 μl of dNTP mixture (2.5 mM of each), 1 μl of each primer (10 pM). This mixture was supplemented with 1 unit of *Taq *DNA polymerase (Fermentas, Lithuania). Forty-two amplification cycles were performed in a T-gradient thermal cycler (Biometra), after an initial denaturation step at 94°C for 5 min. Each cycle consisted of a denaturation step at 94°C for 1 min, an annealing step at 36°C for 1 min, an extension step at 72°C for 1 min. After the last cycle, a final extension step at 72°C for 6 min was performed. The amplification products were analyzed by electrophoresis of 8 μl samples on 2.5% Nu Micropor agarose (PRONA) gels. The above RAPD protocol was established after preliminary trials of various reaction parameters and control experiments to test the reproducibility of this method. It was found that consistently reproducible amplification patterns were obtained only when rigorously optimized and standardized reaction conditions were employed. Discriminatory abilities were studied with several primers: OPA-3, NS-1 [21] and primer-1, primer-6 [22] of different length and G+C content.

**Table 2:** Oligonucleotides and PCR primers used in this study (complementary sequences are underlined).

PCR MP	Restriction	ligated oligonucleotide (POW)	5' CTCACTCTCACCAACAACGTCGAC 3'
	
	enzyme	helper oligonucleotide (HinHELP)	5' AGCTGTCGACGTTGG 3'
	
	*Hin*dIII	primer (POW-AGCTT)	5' CTCACTCTCACCAACGTCGACAGCTT 3'
RAPD	Primer	1247	5' AAGAGCCCGT 3'
	
		1290	5' GTGGATGCGA 3'

### PCR MP procedure

The PCR MP procedure, previously published for *Escherichia coli *differentiation [18], has been optimized and validated for yeasts differentiation using some reference *C. albicans *strains (unrelated) and several related clinical strains from the same hospital ward isolated in a short time (designed as genotype 11 by REA-PFGE in this work).

For the digestion reactions, approximately 40-300 ng of DNA sample was added to 2.5 μl of buffer R (Fermentas, Lithuania), and 0.4 μl (4 U) of the *Hin*dIII endonuclease (Fermentas, Lithuania) (25 μl total volume). After an incubation at 37°C for 15 min, the following ligation mix was added: 1.5 μl of two oligonucleotides (POW and HinHELP [Table 2], 20 pmol of each) that formed an adapter (prepared earlier by 2 min incubation at 70°C, followed by slowly cooling at room temperature for 10 min), 2.5 μl of a ligation buffer (Fermentas, Lithuania), and 0.5 μl of T4 DNA ligase (0.5 U, Fermentas, Lithuania). The samples were then incubated at 22°C for 15 min. The PCR reaction was carried out in a 25 μl reaction mixture consisting of 1 μl ligation solution, 2.5 μl 10× PCR buffer (200 mM Tris-HCl pH 8.5, 100 mM KCl, 100 mM (NH_4_)_2_SO_4_, 20 mM MgSO_4_, 1% Triton X-100), 2.25 μl of a deoxynucleoside triphosphate (dNTP) mixture (2 mM with respect to each dNTP), 0.4 μl (1 U) of *Taq *polymerase RUN (A&A Biotechnology, Poland), and 25 pM of POW-AGCTT primer (Table 2). The denaturation temperature was determined by specific optimization experiments for references and several clinical *C. albicans *isolates, using a gradient thermal cycler (Biometra Tgradient Engine) with a gradient range of 78.0-80.4°C. The PCR reactions were performed as follows: (*i*) 7 min at 72°C to release unligated oligonucleotides and to fill in the single-stranded ends and create amplicons, (*ii*) initial denaturation for 90 s at 78.0-80.4°C gradient across the thermal block, (*iii*) 25 cycles of denaturation for 1 min at 78.0-80.4°C gradient across the thermal block, and (iv) annealing and elongation at 72°C for 2 min 15 s and at 72°C for 5 min after the last cycle.

For all isolates, the PCRs were performed as described above, using the established optimal denaturation temperature of 79.3°C. Electrophoresis of the PCR products, 10 μl out of 25 μl, was carried out on 6% polyacrylamide or 2.5% Nu Micropor agarose (PRONA) gels.

### DNA analysis

The DNA bands, obtained by any of the three methods described above, were visualized by UV transillumination after ethidium bromide staining. Briefly, strains with identical sizes and numbers of well seen bands were considered genetically indistinguishable and assigned to the same type. Strains with banding patterns that differed by up to three bands were considered closely related and described as subtypes, whereas strains with banding patterns that differed by four or more bands were considered to be different types. The patterns obtained from the electrophorograms by any of the three methods used were also converted and analyzed using the Quanty One software, version 4.3.1 (Bio-Rad).

### Reproducibility of the PCR MP

Reproducibility of the PCR MP was tested on ten optionally chosen *C. albicans *isolates (group 2 of strains) and using batches of DNA from two separate DNA extractions amplified in parallel assays and obtained using two extraction methods (Genomic Mini AX Yeast kit - A&A Biotechnology, Poland or GeneMATRIX Yeast DNA Purification kit - Eurx, Poland), employing two different thermal cyclers (a Biometra Tgradient cycler and an Eppendorf MasterCycler EP gradient), and using different *Taq *polymerases (from A&A Biotechnology and Fermentas). Also, the reproducibility of PCR MP patterns was investigated by different persons from another laboratory. PCR products were run in parallel on polyacrylamide gels and digitally recorded.

## Results

### Typing by REA-PFGE

Initially, a total of 123 strains of *C. albicans *were typed with the REA-PFGE method (results not shown). Each pattern consisted of approximately 12-21 fragments. Digestion of the chromosomal DNA with the *Sma*I endonuclease and separation of the fragments by PFGE revealed 26 unique types and 7 subtypes (Table 1). Analysis of the REA-PFGE results for group 2 (patients at the Hematology Clinic of the Public Hospital No. 1 in Gdańsk) revealed a high degree of relatedness between the isolates. The REA-PFGE separated the 89 isolates of *C. albicans *into 14 types with 5 subtypes (6 isolates was not typeable; Table 1). Two genotypes of *C. albicans*, "17" and "15", were markedly predominant, as these were represented by eighteen and fifteen isolates from eleven and fifteen patients, respectively. The REA-PFGE showed that the yeast strains that were isolated to study genotype variation in individual patients were identical when isolated from the same patient (*C. albicans *from patients G1, G7, G8, G21, G23, G24, G31, G36, G40, G48, G53, G54, G55, G68, G71, G72 and G73). Four REA-PFGE types were observed among the 10 *C. albicans *isolates from group 3, the hospital in Szczecin (Table 1).

### Typing by RAPD

The RAPD analysis distinguished 25 unique types with 6 subtypes for *C. albicans *(Table 1). Analysis of the RAPD results for the isolates from the hospital patients (group 2) showed that 63 of the isolates (67.7%) represented four the most common *C. albicans *RAPD types ("17", "9", "19" and "15" contained 18, 16, 15 and 14 isolates, respectively). The remaining thirty isolates (32.3%) belonged to ten other RAPD types, one to eight isolates each.

### Typing by PCR MP

In this study, the PCR MP technique was for the first time used for yeast differentiations. The method applied was similar to the one used for bacterial differentiations in our previous study [18].

**Figure 2:**
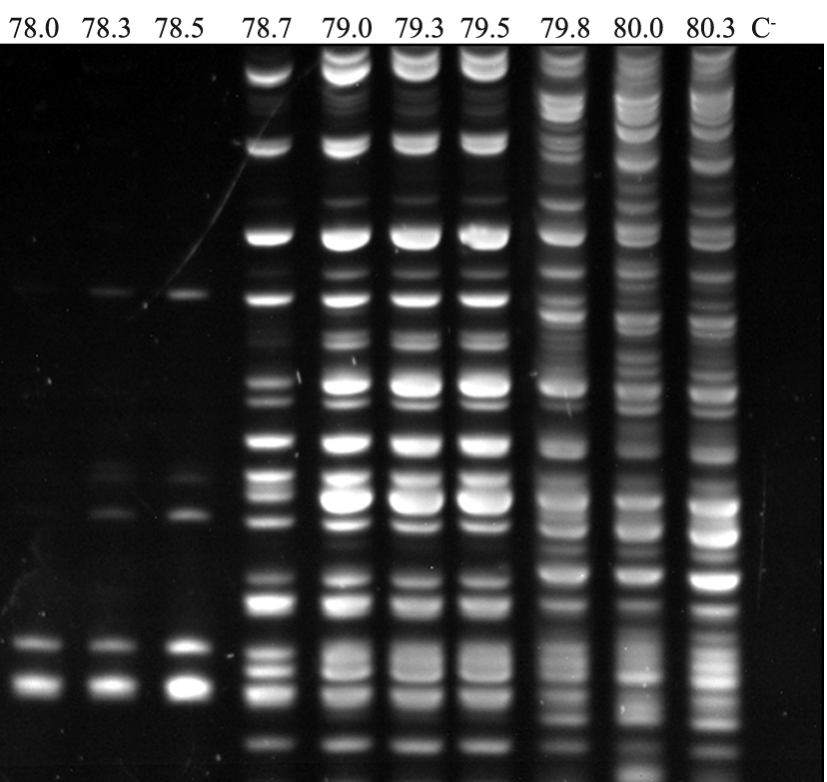
**PCR MPs of *C. albicans *strain at *T*_d _gradients of 78.0-80.3°C**. Electrophoresis of the DNA amplicons were run on 6% polyacrylamide gel.

First, the optimal denaturation temperature for the PCR MP procedure was determined during the optimization experiments with reference strains of *C. albicans *and several clinical isolates, using a gradient thermal cycler (Biometra Tgradient Engine). The optimal denaturation temperature (most stable results in this range) was 79.3°C (Fig. 2).

The discriminatory power of the PCR MP technique usually varies with the restriction enzyme used. Hence, the next step was to determine the discriminatory power of the method using different restriction enzymes for genomic DNA digestion (*Hin*dIII, *Eco*RI, *Nco*I, *Spe*I, *Bgl*II or *Xba*I). In total, DNA from 18 *C. albicans *strains from group one (reference strains and DM collection) was analyzed by PCR MP typing using restriction enzymes indicated above and appropriate adapters (results not shown). The analysis revealed that *Hin*dIII and *Bgl*II enzymes showed the highest discriminatory power. Based on this experiment we chose the *Hin*dIII enzyme for further experiments.

**Figure 3:**
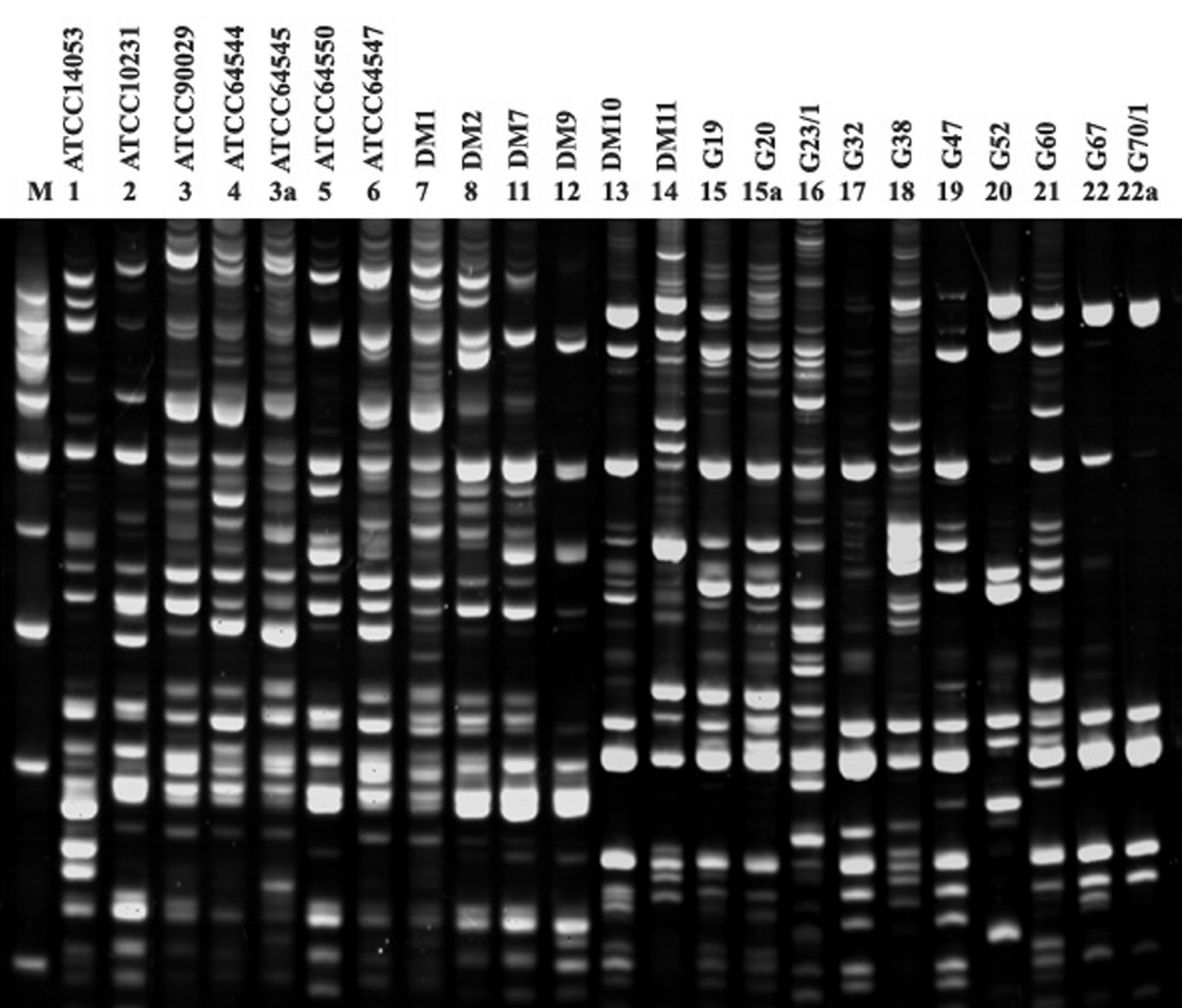
**PCR MP fingerprints of *C. albicans *reference strains and 16 randomly chosen *C. albicans *isolates**. *Hin*dIII restriction enzyme and appropriate adaptors (Table 2) were used. The lane designated M contained the molecular mass marker (1000, 900, 800, 700, 600, 500, 400, 300, 200 bp). The PCR MP fingerprinting type/subtype is given above each lane (ATCC 14053, ATCC 10231, ATCC 90029, ATCC 64544, ATCC 64550, ATCC 64547, DM1, DM2, DM7, DM7, DM9, DM10, DM11, G19, G20, G23/1, G32, G38, G47, G52, G60, G67, G70/1). Electrophoresis of the DNA amplicons were run on 6% polyacrylamide gel.

The PCR MP patterns obtained using the *Hin*dIII enzyme for genomic digestion of *C. albicans *are presented in Fig. 3. Each pattern consists of approximately 15-25 fragments with the size ranging from 100 to 1500 bp.

In total, twenty seven PCR MP types with twenty one subtypes were identified among the *C. albicans *isolates from the three groups (Table 1). All strains from the first group consisting of reference and unrelated strains were determined as different genotypes and proved to be unrelated. Among isolates of second group (76.8% of all strain tested), only fifteen PCR MP types (with seventeen subtypes) were identified, with the most prevalent types/subtypes being "18" and "16". Thirty three *C. albicans *isolates (35.5%) were of those types.

For nineteen patients from the Gdańsk hospital (second group of strains), double *C. albicans *isolates were tested. For sixteen of those patients, their isolates had an identical PCR MP type, whereas isolates from the rest of the patients had closely related subtypes. The identified genotypes were also found in other patients at the Hematology Clinic in the same time. This probably revealed nosocomial transmission of *C. albicans *in that hospital unit.

**Figure 4:**
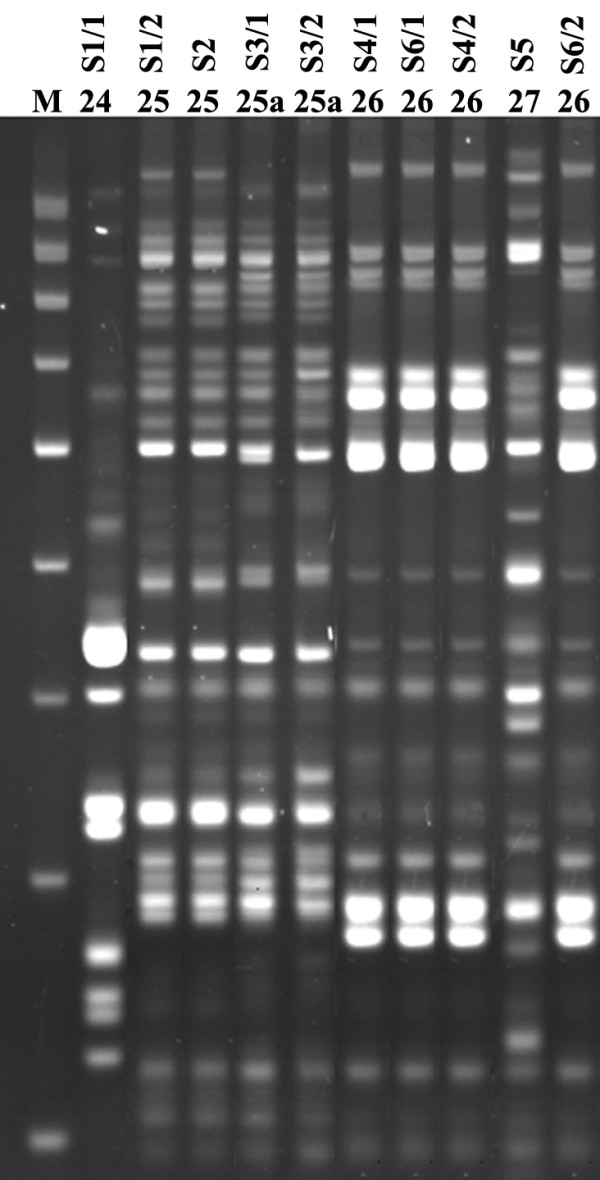
**PCR MP fingerprints for *C. albicans *strains**. The third group, 10 isolates of *C. albicans *from six patients (Table 1). Five of those *C. albicans *strains were isolated from members of one single family (S4, S5 and S6). The lane designated M contained the molecular mass marker (1000, 900, 800, 700, 600, 500, 400, 300, 200 bp). The PCR MP fingerprinting type/subtype is given above each lane. Electrophoresis of the DNA amplicons were run on 6% polyacrylamide gel.

The ability of PCR MP method to differentiate epidemiologically unrelated strains (different geographic origin) was determined by comparison of the typing results for *C. albicans *strains from Gdańsk (second group of strains) and Szczecin (third group of strains). The typing results showed that all strains originating from Szczecin were classified as different genotypes. Also, double isolates from four patients in the Szczecin hospital were analyzed and it was determined that three of patients (S3, S4 and S6) had the same PCR MP type. An analysis of the genotyping results for isolates from members of the same family (patients S4, S5 and S6) revealed that the same genotype ("26") was identified for *C. albicans *isolated from the mother (S4/1) and the child (S6/1) (Fig. 4).

### Reproducibility

We also validated the reproducibility of the PCR MP method. The *C. albicans *isolates were taken from a pure culture to do the PCR MP fingerprinting at two separate occasions. DNA from each culture was isolated using two methods (Genomic Mini AX Yeast kit or GeneMATRIX Yeast DNA Purification kit). The PCR MP fingerprints from each separate run produced identical profiles (results not shown). There was a small variation in the band intensity, but this did not result in any gain or loss of information. PCR MP profiles obtained using two different thermal cyclers (Biometra Tgradient cycler and Eppendorf MasterCycler EP gradient) were found to be somewhat different. However, the grouping of the isolates in types or subtypes was identical (results not shown).

**Figure 5:**
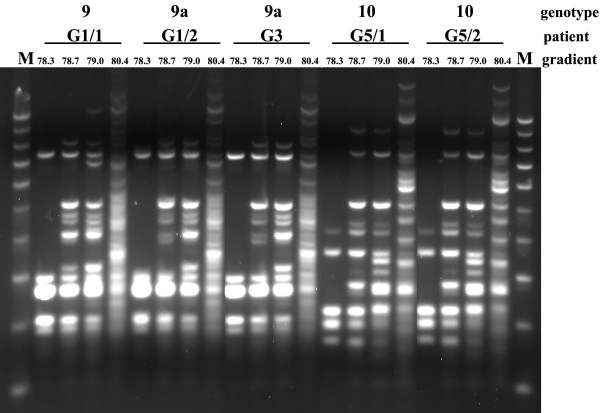
**PCR MP fingerprints of the *C. albicans *at increasing denaturation temperatures in PCR**. Samples were isolated from different clinical specimens of patients (G1 and G5, Table 1) and from various patients (G1, G3 and G5, Table 1). The lane designated M contained the molecular mass marker (1000, 900, 800, 700, 600, 500, 400, 300, 200, 100 bp). Electrophoresis of the DNA amplicons were run on 2.5% agarose gel.

To further ensure that the PCR MP technique has a good reproducibility and satisfactory differentiation efficiency, *C. albicans *isolates from different clinical specimens from two examined patients (G1 and G5, Table 1) and from various patients (G1, G3 and G5, Table 1) were tested with PCR MP at increasing denaturation temperatures. A steady increase in the number of amplified DNA fragments, which was dependent on the denaturation temperature increase, was observed. Despite this, the profiles for isolates belonging to the same genotype were still identical (Fig. 5). Thus the order of DNA band appearance in the PCR performed at subsequently increasing temperatures was constant for a given genomic DNA (genotype).

A good reproducibility of the PCR MP with various *Taq *polymerases was also revealed (results not shown). Results obtained for representative isolates were analyzed by three persons from another laboratory (unaware of the previous classification). Isolates tested were assigned to the same genetic groups by all of us.

### Typeability

The excellent typeabilities of both the RAPD and the PCR MP techniques, as assessed in this study, has been shown by other studies, in which the pooled typeability was 100% for the PCR-based techniques [15,23]. The REA-PFGE technique had a lower typeability (94.2%), which has also been shown by other authors [24,25]. Several problems can occur with this technique: insufficient DNA yield, DNA degradation by endogenous nuclease, and non-complete digestion of the chromosomal DNA.

## Discussion

Typing of candidal strains to determine their genetic relatedness has been done by a variety of phenotypic and genetic techniques, including physiochemical tests, determination of antigenic differences, and the use of molecular biology based methods for comparison of DNA profiles. However, no "gold standard" method has been identified for determination of candidal strain relatedness. In the present study, the usefulness of the PCR MP technique for yeast strain differentiation was demonstrated, and the results compared with those obtained with the REA-PFGE and RAPD methods. When analyzed with three different genotyping methods, the 123 *C. albicans *strains yielded 26 different REA-PFGE types (with 7 subtypes), 25 RAPD different types (with 6 subtypes), and 27 PCR MP types (with 21 subtypes) (Table 1). For using 18 unrelated *C. albican*s isolates (group one, reference strains and DM collection), we distinguished fourteen types (with two subtypes) for the REA-PFGE method, fourteen types (with one subtype) for the RAPD analysis and fourteen types (with 2 subtypes) for the PCR MP technique. For each of the three methods, REA-PFGE, RAPD and PCR MP, both benefits and drawbacks have been described. Comparative studies have shown that the RAPD analysis is a faster and less technically demanding methodology [24,26]. Substantially smaller amounts of purified DNA (*i.e*. 20-50 ng) are required compared with the amount needed to do REA-PFGE. However, as previously demonstrated [26-28] the reproducibility of the RAPD analysis is dependent upon a careful standardization of the PCR conditions (especially choice of the primer, DNA and primer concentration, polymerase type or thermal cycler profile are critical factors in producing informative patterns). The REA-PFGE technique requires larger amounts of DNA, but has proven to be extremely reproducible [27]. The PFGE analysis is sometimes considered to be the most difficult method to work with, requiring intact chromosomes and specialized electrophoresis equipment, and is also the most time-consuming. Despite this, others think that this is a highly sensitive and useful technique for strain discrimination [28]. Due to the mentioned drawbacks, the REA-PFGE is not an ideal typing method for health departments undertaking routine analysis of large numbers of isolates. The PCR MP is a much easier analysis than the REA-PFGE, as it does not require intact chromosomes and specialized electrophoresis equipment, and it is also less time-consuming. Another advantage is that only a limited amount of DNA (40-1000 ng) is needed since the fragments are PCR amplified with the same thermal profile. Besides, in contrast to RAPD, the broad range of DNA amounts can be used in PCR MP reaction without any influence on amplification pattern. Since stringent annealing temperatures in PCR MP are used during the amplification (only DNA fragments with adapters are amplified), the technique is more reproducible and robust than other methods, such as the RAPD, in which annealing temperatures should be optimized for each primer used. However, the amplification performed at stringent annealing temperature in PCR MP is closely dependent on the thermal stability of DNA-fragments obtained (only completely denatured fragments are amplified). Based on the results in the present study, we conclude that the PCR MP technique has a similar discriminatory power and a higher reproducibility, than the RAPD method. The results also suggest that the power of discrimination is similar to the one obtained with the REA-PFGE technique for yeast differentiation. Comparative studies of typing methods have shown that the RAPD can provide a fast, economical and reproducible means of typing *C. albicans *isolates, with a level of discrimination approaching that of the REA-PFGE [29]. As for the REA-PFGE and RAPD methods, the advantage of the PCR MP analysis is that the patterns are a representation of the whole genome.

In contrast to the REA-PFGE and RAPD, the PCR MP typing method allows the possibility to increase the number of DNA restriction fragments amplified by increasing the denaturation temperature during the PCR. A steady increase in the number of DNA fragments amplified is dependent on the denaturation temperature increase. It is especially important when typing closely related isolates.

There is one significant drawback of the PCR MP method, which is known to be sensitive to small fluctuations in temperature. For that reason, validation of the thermal cycler is an important issue for the generation of reliable and repeatable data.

## Conclusion

The aim of the current study was to evaluate the PCR MP typing method for the differentiation of *C. albicans *strains. It is the first time that the recently developed genotyping PCR MP technique, described by Masny and Plucienniczak [17] and Krawczyk *et al. *[18], has been used for *C. albicans *strains typing. Using the PCR MP, it was possible to group isolates recovered from the same patient and from different patients, both visually and with the aid of computer-assisted procedures (program computing). However, it is no possible to create the base of data of analyzed strains to compare the data between laboratories as it is possible with MLST. With this technique it was also possible to differentiate highly related isolates (isolates from the same patient and patients attended in the same hospital ward). We thus conclude that the optimized PCR MP procedure may offer a discriminatory method for genotyping of yeasts in epidemiological studies, as well as in the control of nosocomial infections. Considering the low costs and relatively high discriminatory power of the PCR MP method, we propose that this is a better choice for epidemiological studies of candidial strains than the use of the PFGE analysis of chromosomal macrorestriction fragments obtained with several restriction enzymes in large-scale hospital studies of intra-species genetic relatedness of *C. albicans *strains.

Probably, the described here PCR MP method can be successfully used also for epidemiological studies, especially in short period of time, of other medically important fungi.

## Competing interests

The authors declare that they have no competing interests.

## Authors' contributions

BK participated in the design of the study and coordination, carried out the genetic typing and drafted the manuscript; JL-C carried out the genetic typing, participated in its design and helped to draft the manuscript; AM and MN carried out the genetic typing; JK corrected and edited the final version of manuscript. All authors read and approved the final manuscript.

## Pre-publication history

The pre-publication history for this paper can be accessed here:

http://www.biomedcentral.com/1471-2334/9/177/prepub
